# A Framework for Weather-Driven Dengue Virus Transmission Dynamics in Different Brazilian Regions

**DOI:** 10.3390/ijerph18189493

**Published:** 2021-09-09

**Authors:** Leon Diniz Alves, Raquel Martins Lana, Flávio Codeço Coelho

**Affiliations:** 1 Centro Federal Celso Suckow da Fonseca, Rio de Janeiro 20271-110, Brazil; leon.alves@cefet-rj.br or; 2 Computational Biology and Systems, Oswaldo Cruz Foundation, Rio de Janeiro 21040-900, Brazil; 3 Scientific Computing Program, Oswaldo Cruz Foundation, Rio de Janeiro 21040-900, Brazil; raquelmlana@gmail.com or; 4 School of Applied Mathematics, Getulio Vargas Foundation, Rio de Janeiro 22250-900, Brazil; 5 Institute of Global Health, University of Geneva, 1205 Geneva, Switzerland

**Keywords:** dengue, transmission model, *Aedes aegypti*, environment variables, tropical diseases

## Abstract

This study investigated a model to assess the role of climate fluctuations on dengue (DENV) dynamics from 2010 to 2019 in four Brazilian municipalities. The proposed transmission model was based on a preexisting SEI-SIR model, but also incorporates the vector vertical transmission and the vector’s egg compartment, thus allowing rainfall to be introduced to modulate egg-hatching. Temperature and rainfall satellite data throughout the decade were used as climatic model inputs. A sensitivity analysis was performed to understand the role of each parameter. The model-simulated scenario was compared to the observed dengue incidence and the findings indicate that the model was able to capture the observed seasonal dengue incidence pattern with good accuracy until 2016, although higher deviations were observed from 2016 to 2019. The results further demonstrate that vertical transmission fluctuations can affect attack transmission rates and patterns, suggesting the need to investigate the contribution of vertical transmission to dengue transmission dynamics in future assessments. The improved understanding of the relationship between different environment variables and dengue transmission achieved by the proposed model can contribute to public health policies regarding mosquito-borne diseases.

## 1. Introduction

Dengue is a viral mosquito-borne disease that has four serotypes (DENV-1, DENV-2, DENV-3 and DENV-4) and is transmitted by *Aedes aegypti* [1] and *Aedes albopictus* [2] mosquitoes. Dengue is an endemic disease in many countries worldwide, displaying marked seasonality. The ecology of the DENV vector has been widely studied and modelled taking into account temperature-dependency, which is regarded as the main seasonality driver of this disease [3,4,5]. Deterministic models, such as the SIR/SEIR (Susceptible (S), Exposed (E), Infectious (I) and Recovered (R)) model have often been employed to model DENV transmission dynamics [6,7,8]. In these transmission models, the explicit inclusion of the vector population compartment is usually represented by the adult stage, which is the stage responsible for transmitting the DENV virus to humans [9,10]. This representation limits the ability to study the effects of certain environmental factors on specific immature stages of the vector. Mosquito vertical transmission, for example, requires an explicit egg compartment of the model that can incorporate egg longevity in the environment, allowing them to act as a long-term virus reservoirs.

Existing DENV transmission models vary substantially [11,12], but many recognize that environmental fluctuations are key to understanding mosquito population dynamics. Temperature, for example, modulates oviposition, survival rates, biting rates and the extrinsic incubation period of DENV [13,14,15], whereas rainfall is an egg hatching trigger, as it provides oviposition breeding sites and the development of the mosquito’s aquatic stages [16]. These environmental aspects contribute to mosquito populations displaying strikingly seasonality and geographically distribution between tropical and subtropical regions, such as Brazil [17].

Huber et al. [7] demonstrated that a SEI-SEIR model including human and vector compartments with temperature dependence can be implemented in such a way that transmission is more effective at higher temperatures and decreases at lower temperatures. Rainfall is also an important component when predicting dengue incidence, although most models do not include this weather variable [6,7]. Including rainfall in any model by itself is a challenge, as eggs can survive for months during dry seasons [18]. On the other hand, excessive and prolonged rain may wash out larvae from breeding sites [16]. Moreover, a lag between the beginning of the rainy season and increasing dengue incidence is also observed [19].

In the present study a new model based on Huber et al. [7] is proposed. This new model also includes temperature-driven biological responses related to DENV transmission dynamics, while also adding an egg compartment to the *Aedes* population, thus allowing rainfall to be introduced as a modulating variable in egg-hatching. Vertical transmission of DENV has been demonstrated in the laboratory for *Ae. aegypti* and *Ae. albopictus* [20] and has the potential to sustain endemic transmission in the long term [21], so it is featured in the model as well.

Satellite-based temperature and rainfall data, as well as reported dengue incidence from the Brazilian Unified Health System (SUS) were obtained for four municipalities from January 2010 to December 2019 to be used in case studies. Brazil has experienced seasonal dengue epidemics since 1986, registering the largest epidemics of all American continent countries [22]. In 2019 alone, the country reported over 2 million cases [23]. All four DENV serotypes circulate in Brazil, and the main vector is the *Ae. aegypti* mosquito, found throughout the entire country [22]. Therefore, the following questions are addressed in this work: (1) How is the dynamics of DENV transmission modulated by weather variables? and (2) Is vertical mosquito transmission a significant mechanism for the long-term persistence of DENV in a certain area?

## 2. Materials and Methods

**Figure 1:**
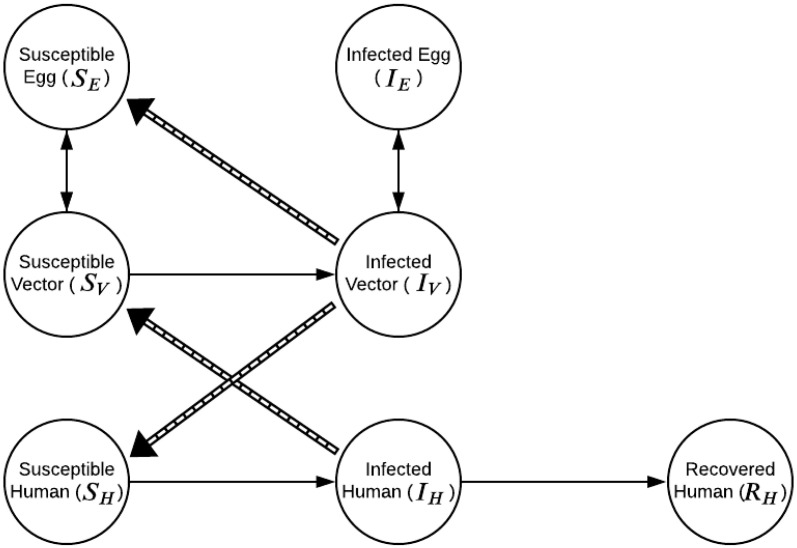
SI-SI-SIR model compartments. $S_{E}$, $I_{E}$, $S_{V}$ and $I_{V}$, $S_{H}$, $I_{H}$ and $R_{H}$ represent the non-infected and infected eggs of the vector population; the susceptible and infectious compartments of the adult mosquito population; and the susceptible, infectious and recovered portions of the human population, respectively. Solid arrows indicates transmission direction.

Our study proposes a model, implemented as a system of ordinary differential equations, to describe the dynamics of DENV transmission in eggs (SI), adult mosquitoes (SI) and humans (SIR). The model, henceforth denoted SI-SI-SIR, does not include recovered compartments for *Ae. aegypti* populations, as mosquitoes are assumed to remain infected for their entire life [24]. Figure 1 presents the model diagram.

The following Equations (1) explain the interactions between each population compartment.

(1a) $$\begin{array}{cc} \frac{dS_{E}}{dt} & =o\left(T\right)S_{V}+\left(1-v_{t}\right)o\left(T\right)I_{V}-\left(\mu_{e}+d\left(R\right)\right)S_{E} \end{array}$$

(1b) $$\begin{array}{cc} \frac{dI_{E}}{dt} & =v_{t}o\left(T\right)I_{V}-\left(\mu_{e}+d\left(R\right)\right)I_{E} \end{array}$$

(1c) $$\begin{array}{cc} \frac{dS_{V}}{dt} & =d\left(R\right)f_{v}s_{a}\left(T\right)S_{E}\left(1-\frac{N_{V}}{KN_{H}}\right)-\left(a\left(T\right)p_{hm}\left(T\right)\frac{\left(I_{H}+i_{m}\right)}{N_{H}}+\mu_{v}{\left(T\right)}^{-1}\right)S_{V} \end{array}$$

(1d) $$\begin{array}{cc} \frac{dI_{V}}{dt} & =d\left(R\right)f_{v}s_{a}\left(T\right)I_{E}\left(1-\frac{N_{V}}{KN_{H}}\right)+\left(a\left(T\right)p_{hm}\left(T\right)\frac{\left(I_{H}+i_{m}\right)}{N_{H}}\right)S_{V}-\mu_{v}{\left(T\right)}^{-1}I_{V} \end{array}$$

(1e) $$\begin{array}{cc} \frac{dS_{H}}{dt} & =u_{h}N_{H}-a\left(T\right)p_{mh}\left(T\right)I_{V}\frac{S_{H}}{N_{H}}-\mu_{h}S_{H} \end{array}$$

(1f) $$\begin{array}{cc} \frac{dI_{H}}{dt} & =a\left(T\right)p_{mh}\left(T\right)I_{V}\frac{S_{H}}{N_{H}}-\left(\gamma +\mu_{h}\right)I_{H} \end{array}$$

(1g) $$\begin{array}{cc} \frac{dR_{H}}{dt} & =\gamma I_{H}-\mu_{h}R_{H} \end{array}$$

The Equation (1a) and Equation (1b) represent the egg population, where $S_{E}$ comprises the susceptible egg compartment (1a) and $I_{E}$, the infected eggs (1b). o(T) represents the number of eggs laid by female mosquitoes as a function of temperature. d(R) comprises the egg development rate as a function of rainfall. $f_{v}$ is the female portion of the population, $v_{t}$ is the vertical transmission rate and $\mu_{e}$ is the egg mortality rate. Temperature and rainfall time series are denoted, respectively, by *T* and *R*.

Equation (1c) and Equation (1d) represent adult mosquitoes with $S_{V}$ standing for susceptible (1c) and $I_{V}$, for infected adult mosquitoes (1d). $s_{a}\left(T\right)$ comprises the egg-to-adult temperature-dependent survival rate and $\mu_{v}\left(T\right)$, the adult temperature-dependent mortality rate. The aquatic phase (larvae and pupae) is not explicitly represented in the model and, therefore, survival and mortality rates are absorbed into the egg-to-adult rates described above. $N_{V}$ represents the total vector population size ($S_{v}+I_{v}$). *K* is the environmental carrying capacity, which constrains the growth of the mosquito population. a(T) is the mosquito biting rate, $p_{hm}$ is the probability of a human infecting a mosquito per bite, $i_{m}$ is the rate of pendular immigration (defined below) of infected humans and $N_{H}$ consists in the total number of humans in the model ($S_{H}+I_{H}+R_{H}$).

The SIR submodel (Equation (1e), Equation (1f) and Equation (1g)) represents a human population with compartments $S_{H}$, $I_{H}$ and $R_{H}$ governed by (1e), (1f) and (1g). The human population was assumed as constant, i.e., same birth and death rates ($\mu_{h}$) plus a small pendular migration rate, defined as residents that inhabit one municipality and work or study in another [25]. Pendular migration does not affect the model population size, but does affect the infection force, as mosquitoes can bite infected humans originated from other areas. γ represents the human recovery rate after infection. Finally, $p_{mh}\left(T\right)$ comprises the probability of a mosquito infecting a human by biting.

This model exhibits a time resolution of 8 days instead of the commonly applied daily resolution, due to the time resolution of the employed satellite data of 8 days. Because of this, the exposed compartment, originally present in the model developed by Huber et al. [7], was removed for the mosquito and human population in the proposed model.

### 2.1. Initial Conditions

The constant model parameters are presented in Table 1. The sex-ratio of the mosquito population was assumed to be 1:1 [26]. Egg and adult mosquito mortality rates were obtained from the literature as daily time resolution and converted to 8 days. Human Birth and mortality rates were based on 2010 data [27] considering the 8-day average. As an initial condition, this study assumed a 10% vertical transmission rate from female mosquitoes to offspring, but also evaluated transmission values from 0% to 20%, as the literature points to substantial vertical transmission rate variability [28,29,30].

Concerning carrying capacity *K*, Neira et al. [31] reported a ratio of 0.7 female mosquitoes per person in a given area, and, as this value is in accordance with other studies [32], it was employed herein as the initial carrying capacity value (Figure S1 in Supplementary Materials File S1). Finally, 0.1% of the total number of immigrants (or tourists) were considered infected, which still allows for effective reinvasion.

The temperature- and rainfall-dependent parameters are given in Table 2. Temperature-dependency is represented by a Brière or quadratic function fitted in previous studies employing experimental laboratory data [34]. Brière is a function that assumes lower and upper thresholds, asymmetry concerning the optimum parameter, and a sharp decline in values above optimum parameters (Figure S2 in Supplementary Materials File S1); in contrast to a quadratic function, where symmetry concerning the optimum parameter is observed [35]. Mordecai et al. [34] applied these functions to calculate a minimum ($T_{min}$) and maximum, ($T_{max}$) temperature, and a constant rate (*c*) for each temperature-driven rates. The rainfall-dependent variable consists in the egg hatching rate, d(R). The mean hatching rate reported by Alto and Juliano [36] was applied to a quadratic function (Figure S3 in Supplementary Materials File S1). This fit reflects a positive correlation between egg hatching and rainfall, although excessive rainfall volumes can lead to negative effects, as they may cause a flushing event, emptying mosquito breeding sites [16].

The initial value for a susceptible human population ($S_{H}\left(0\right)$) was determined empirically when fitting the model to the data. $I_{H}\left(0\right)$ was set as the dengue prevalence for the first week of January 2010, comprising the beginning of the analyses. The number of tourists was provided by the Ministry of Tourism [37]. The total number of tourists in 2010 was converted to an 8 days time resolution by multiplying the value by $\frac{8}{365}$. $R_{H}\left(0\right)$ was set to $N_{H}\left(0\right)-S_{H}\left(0\right)-I_{H}\left(0\right)$.

The adult mosquito populations were directly proportional to the human populations according to the 0.7 mosquitoes per person ratio reported by Neira et al. [31]. Thus, $S_{V}\left(0\right)$ and $I_{V}\left(0\right)$ were directly proportional to $S_{H}\left(0\right)+R_{H}\left(0\right)$ and $I_{H}\left(0\right)$, respectively, multiplied by 0.7. This ratio was also used for the egg compartment, where $S_{E}\left(0\right)$, the initial state of susceptible eggs, was equal to $0.7(S_{H}\left(0\right)+R_{H}\left(0\right))$, while $I_{E}\left(0\right)$, the initial state of infected eggs, was set as $0.7I_{H}\left(0\right)$.

### 2.2. Case Studies

**Figure 2:**
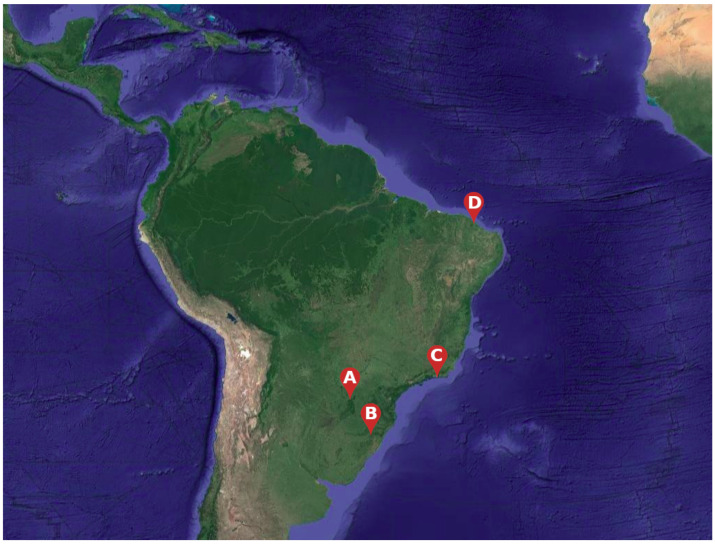
Brazilian municipalities as noted in a South America satellite view: (A) Foz do Iguaçu; (B) Porto Alegre; (C) Rio de Janeiro; (D) Fortaleza [47].

Four Brazilian municipalities with different DENV transmission dynamics and environmental patterns were chosen to evaluate the model, namely Rio de Janeiro, Fortaleza, Foz do Iguaçu and Porto Alegre (Figure 2). All of them, except for Porto Alegre, have suffered multiple severe dengue outbreaks between 2010 and 2019 [38].

Fortaleza, the capital of the state of Ceará, is located on the northern coast of Northeastern Brazil, near the equator line. It exhibits a high demographic density of 8390.76 people/km^2^, with a population size of 2,452,185 inhabitants according to the 2010 Census [39]. Fortaleza displays a warm and sub-humid tropical climate [40], with an average temperature of 29 ± 1.4 ${}^{{}^{\circ}}$C in the last decade [41]. Periods of intense droughts with occasional rains are common; with an average rainfall of 14 ± 21 mm for an 8-day cycle in the last decade [42], the lowest average rainfall levels of all municipalities considered in this assessment. Due to its tropical climate and high population density, it is highly receptive to infestation by *Ae. aegypti*. Although it is a coastal area, Fortaleza does not receive the same influx of tourists as other municipalities in Brazil [37].

The municipality of Rio de Janeiro, the capital of the state of Rio de Janeiro, also exhibits a high demographic density, of 5556 people/Km^2^, and a population size of 6,320,446 inhabitants [39]. It shares some weather characteristics with Fortaleza, with a tropical humid and warm climate weather [43], with an average temperature of 26 ± 3 ${}^{{}^{\circ}}$C in the last decade [41]. Occasionally, winters can comprise warm weeks with an average temperature of 30 ${}^{{}^{\circ}}$C [43]. Average rainfall values from last decade indicate 18 ± 19 mm per an 8-day cycle [42], characterizing dry weather, although with heavy rains, especially in the fall.

Foz do Iguaçu is located in western Paraná, in southern Brazil. It is set at an altitude of 164 m, with a demographic density of 418.5 people/Km^2^, and a population size of 256,088 inhabitants [39]. The climate is subtropical humid mesothermal, with an average temperature of 24 ± 4 ${}^{{}^{\circ}}$C [41] and average rainfall of 26 ± 25 mm [42] in the last decade. The municipality shares borders with two countries, Paraguay and Argentina, an obligatory trade route stop between these countries [44]. At the same time, it is known by its tourist activities [45]. Therefore, in spite of the fact that it has a relatively small resident population and is located far from the coast, Foz do Iguaçu receives thousands of tourists every day [37].

Porto Alegre, the capital of the state of Rio Grande do Sul, is the southernmost municipality among those analyzed in this study. It displays a humid subtropical climate [46], and is located on the state coast, similarly to Rio de Janeiro and Fortaleza. Porto Alegre has a demographic density of 2837.52 people/Km^2^ and a population size of 1,409,351 inhabitants [39]. It receives thousands of tourists from Argentina and Uruguay, that border Rio Grande do Sul [37]. Porto Alegre is the coldest municipality considered herein, averaging 21 ± 4 ${}^{{}^{\circ}}$C in the last decade [41]. The average rainfall per 8 days is 22 ± 20 mm [42] during the same period.

### 2.3. Data

#### 2.3.1. Epidemiological Data

Dengue notifications are mandatory in Brazil and were obtained from the InfoDengue API [38]. InfoDengue provides a reported incidence of dengue per municipality per week. The obtained incidences were transformed to daily resolution by dividing the values per 7. Subsequently, the values for an eight-day period were summed to match the periodicity of the satellite-based weather data.

#### 2.3.2. Temperature Data

The Earth Surface Temperature (LST) data were obtained from the MODerate Resolution Imaging Spectroradiometer (MODIS / MOD11A2) sensor on the Terra satellite [41], which exhibits an 8-day Emissivity and a 1 square km Sine Grid resolution. The satellite data emitted refer to the average daily (LST Day) and night (LST Night) temperatures every 8 days. The averages between day and night temperatures were employed for all pixels within the municipality borders to obtain the average for the entire municipality (Figure S4, Supplementary Materials File S1).

#### 2.3.3. Rainfall Data

Rainfall data were obtained from the Climate Hazards Group InfraRed Precipitation with Station Data (CHIRPS) database at the Climate Hazard Center belonging to the University of California, Santa Barbara (UCSB-CHG) [42]. The CHIRPS has operated for over 30 years, with a daily resolution of 5 km. Rainfall data was obtained per municipality using the same method described for temperature (Figure S5, Supplementary Materials File S1).

Initial model conditions for each municipality are displayed in Table 3.

### 2.4. Model Calibration

A Sobol sensitivity analysis was conducted to determine the leverage of each parameter concerning the fit between the simulated and observed data [48]. The sensitivity analysis identified the parameters that more substantially affect model adherence to the observed data. The Python Sensitivity Analysis Library (SALib) was used [49]. The sum of squared errors (SSE) between the simulated and observed time series was applied as the model output. The following parameters and respective ranges were scanned in this analysis: o(T) (0.5–2), $v_{t}$ (0–0.3), *K* (0.5, 3), a(T) (0.5–2), $i_{m}$ (0.00001–0.01), $p_{mh}$ (0.5–2), $p_{hm}$ (0.5–2), $f_{v}$ (0.3–0.7), $u_{e}$ (0.01–0.15), $u_{v}$ (0.3–0.7), γ (0.4–1.6), $u_{h}$ (0.00001–0.001), $s_{a}\left(T\right)$ (0.5–2). For constant parameters, the range represents the exact variable value; for dependent parameters, the range represents a multiplier applied to the constant *a* in the Brière $\left[aT\left(T-b\right){\left(c-T\right)}^{\frac{1}{2}}\right]$ and quadratic [a(T−b)(T−c)], $\left[a\left(R^{2}+bR\right)\right]$ functions.

Following the sensitivity analysis, constant and climate-dependent parameters obtained from the literature were adapted for each municipality, in order to better adapt to the climate context of each municipality. This process was performed empirically, observing the analysis result. We sought to alter the parameters as little as possible from those reported in the literature, and even with the employed adaptations, the same order of magnitude of the original values was always maintained. The adaptations were performed based on the understanding that mosquitoes adapt differently to the climate of each municipality, maintaining all remain biologically realistic, as slightly different biological parameters than those observed in the laboratory under controlled and fixed conditions are, therefore, possible and likely.

## 3. Results

### 3.1. Sensitivity Analysis

**Figure 3:**
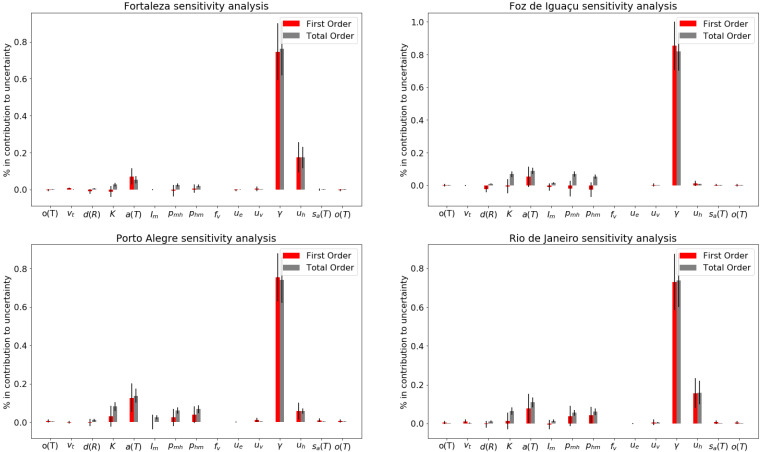
Sensitivity analysis for each model parameter. From top to bottom, from left to right: Fortaleza, Foz do Iguaçu, Porto Alegre and Rio de Janeiro.

According to the sensitivity analysis, the dengue recovery rate (γ), human birth and mortality rate ($\mu_{h}$) and biting rate (a(T)) affect the model behavior more strongly than the other evaluated parameters. When averaging the values for the four municipalities, the following parameters had first and total order sensitivity coefficients higher than 5%: γ, $\mu_{h}$ and a(T) (Figure 3). The second order sensitivity coefficients revealed that mosquito-to-human infection probability per bite ($p_{mh}\left(T\right)$), human-to-mosquito infection probability per bite ($p_{hm}\left(T\right)$) and carrying capacity (*K*) exhibited dependent associations between each other and the other assessed parameters (Figures S6–S9, Supplementary Materials File S1). The recovery rate (γ) was the only parameter that exhibited a negative correlation with SSE ($R^{2}>80\%$) for all municipalities (Figures S10–S13, Supplementary Materials File S1). The other evaluated parameters exhibited weak correlations with SSE, less than 50% and greater than −50% for the Pearson Correlation Index. When comparing parameter sensitivities between municipalities, Rio de Janeiro was the only municipality where $\mu_{v}\left(T\right)$ comprised over 0.5% of the total order sensitivity coefficient, while Fortaleza was the only municipality not sensitive to $i_{m}$, the rate of infectious immigrants.

### 3.2. Calibration Process

The SI-SI-SIR model was qualitatively evaluated under four weather regimes across different Brazilian municipalities. Starting with parameter values from the literature (Table 1 and Table 2), the vicinity of their values was explored to see how they would affect the model’s fit to data. These changes aimed at bringing the parameters values closer to representing local mosquito population while keeping values in the same order of magnitude of original values (Table 4). The constant coefficients to the carrying capacity (*K*), biting rate (a(T)), development rate (d(R)) and the probabilities of transmission between human and mosquito ($p_{mh}\left(T\right)$ and $p_{hm}\left(T\right)$) were adapted for each municipality.

The most significant changes in this qualitative process were observed in the municipality of Fortaleza, as, similarly to Foz do Iguaçu, the carrying capacity was 5-fold higher than that of Rio de Janeiro and Porto Alegre. Furthermore, d(R) and $p_{hm}\left(T\right)$ were reduced to a third. Foz do Iguaçu’s $p_{mh}\left(T\right)$ was also 50% lower, while d(R) decreased to 5%. Porto Alegre variables displayed at least a half decrease, while Rio de Janeiro exhibited small changes to d(R) and *K*.

**Figure 4:**
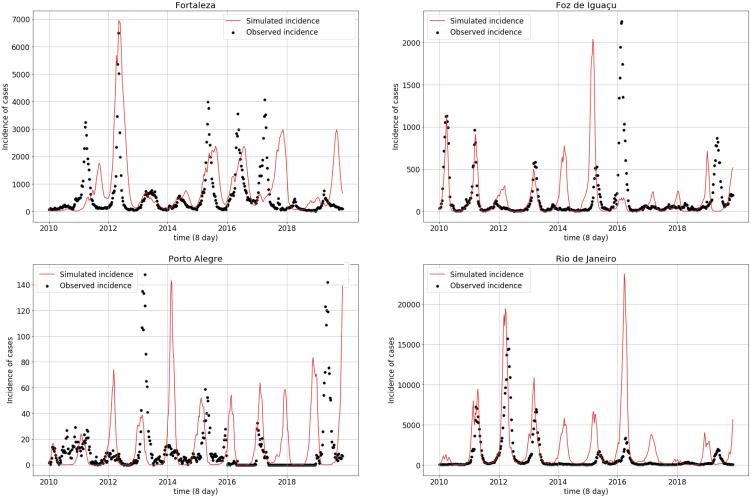
Observed (black dots) and simulated (red line) dengue incidence time series for the four studied municipalities.

Simulations obtained with the parameter values from Table 4 are displayed in Figure 4. Fortaleza and Rio de Janeiro most often presented simulated epidemic peaks resembling the observed peaks. This contrasts with Porto Alegre and Foz do Iguaçu, which exhibited less agreement between the simulated and observed peaks. From 2010 to 2016, simulations adhered to the observed series, both in terms of peak magnitudes and seasonality, while the simulated incidence began to stray away from the observations both in magnitude and in phase in all cities after 2016.

Table 5 indicates the attack rates for each municipality, i.e., the sum of all infected individuals during the period divided by the size of the population at risk. This differed between the simulated and observed time series. Foz do Iguaçu presents a lower simulated attack rate than the observed (9.64% difference), while epidemic overestimation in 8.05%, 6.02% and 0.09% were observed for Rio de Janeiro, Fortaleza and Porto Alegre respectively.

### 3.3. Vertical Transmission

**Figure 5:**
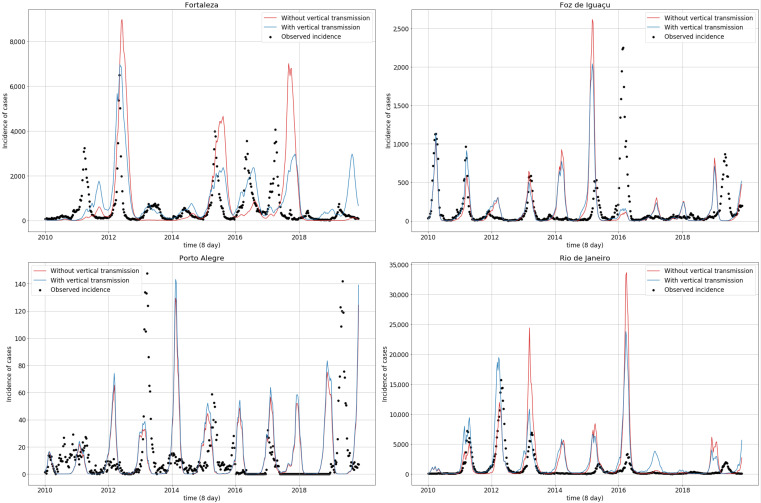
Observed (dots) and simulated (solid line) dengue incidence time series, with (blue lines) and without (red lines) vertical transmission, for the four studied municipalities. Parameter values used in the simulations are shown in Table 4.

Figure 5 compares simulations with and without vertical transmission. Removing vertical transmission influenced the epidemic peak size. Foz do Iguaçu and Rio de Janeiro, for example, exhibited a minor incidence peak in 2011, which have increased consistently since 2013, and sometimes, as in 2015, presented higher differences. In Fortaleza, removing vertical transmission increased dengue incidence in certain epidemic peaks, especially in 2013. Porto Alegre did not exhibit significant epidemic size variations, although vertical transmission smoothed the epidemic peaks.

**Figure 6:**
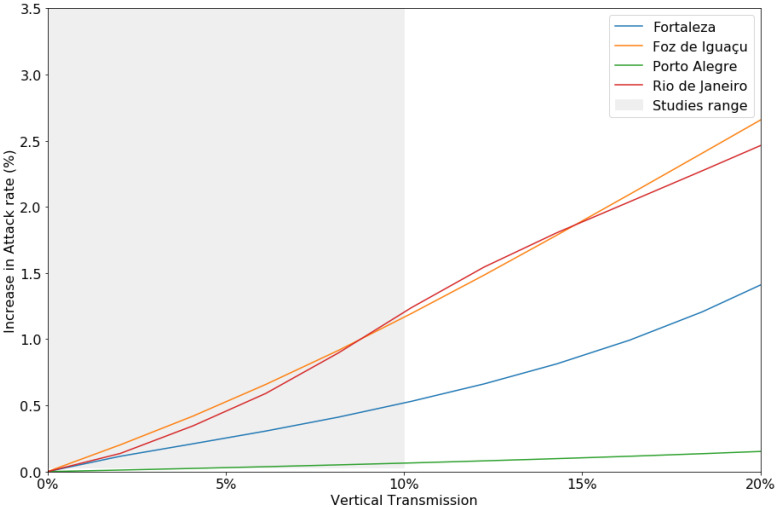
Increased attack rates (%) with different vertical transmission levels. The gray area indicates the range of vertical transmission reported in the literature.

Figure 6 indicates the influence of vertical transmission on attack rates. Vertical transmission was positively correlated with attack rates for every municipality, indicating that with increasing vertical transmission accompany higher attack rates. In Rio de Janeiro, increasing the vertical transmission value from 3% to 13% [28,29,30] caused a 2% disparity in attack rates between the simulations, indicating an 126,408 increase in dengue cases. Porto Alegre, on the other hand, requires a vertical transmission success rate of over 50% to begin displaying epidemic behavior.

## 4. Discussion

This work proposed and analyzed a transmission model to represent the dynamics of DENV transmission. It was validated employing data from 2010 to 2019 in four Brazilian municipalities. The model provides a framework for understanding weather-driven dengue transmission considering rainfall dependency, one of the challenges for these type of models [50,51]. The addition of susceptible and infected egg states allowed for the inclusion of rainfall dependency and vertical transmission in the studied model.

This work was inspired by the model developed by Huber et al. [7] whose performance was tested using different sinusoidal functions to represent temperature. Here, we used temperature and rainfall time series derived from weather satellite data. Other works in the literature usually employ sinusoidal functions to represent temperature variations [7,50,52,53] or constant temperature [24,54,55]. Our model was capable of producing similar patterns to those observed for dengue incidence, presenting both seasonality and similar epidemic sizes. In contrast to Huber et al. [7], our simulations displayed sharper epidemic peaks, more similar to observed peaks, due to the use of observed temperature data and the addition of the egg compartment, which drove the force of infection even higher, as adult mosquitoes are almost always at the carrying capacity level when the temperature permits.

Environmental variables affected the dynamics, as expected: in Fortaleza, Rio de Janeiro and Foz do Iguaçu, that display warmer climates where transmission is facilitated, both the simulated and observed time series exhibited higher dengue incidence and the same epidemic pattern. In Porto Alegre, a municipality exhibiting a colder climate, a lower dengue incidence was observed, not enough to generate epidemic cycles, instead producing only sporadic outbreaks. Nonetheless, the qualitative calibration process mostly affected parameters which, in the literature, are highly variable. The colder weather of Porto Alegre represents unfavorable conditions for the vector, which explains the fact that most parameter values are smaller than in other cities. While Fortaleza and Foz do Iguaçu present favorable climates, allowing for higher carrying capacity leading to a better fit to data. Lastly, the sensitivity analysis clarified how each parameter affected the model’s ability to match the observed dynamics. It further demonstrated that the parameters, especially dengue recovery rate (γ), can be better adapted to the Brazilian environment, since a higher γ reduced the SSE.

With respect to the discordance between the simulated and observed time series, most of the inaccuracies concerning epidemic size and peak dates were noted after 2016, which can be explained by the following factors: (a) the model conflates all dengue serotypes into only one, and though this simplification is common in conceptual framework articles, it does not adequately represent the susceptible population, which can accumulate errors over time; (b) after 2016, Zika and chikungunya were introduced in the studied municipalities (Figures S14 and S15 in Supplementary Materials File S1), and this disease co-circulation can lead to their misdiagnosis, as they result in similar clinical conditions [56]; (c) in 2015–2016, extreme weather changes due to the El Niño phenomenon were observed [57], which may cause changes in epidemiology scenarios [58,59,60]; (d) not all developmental *Ae. aegypti* stages were accounted for in the model, with the aquatic phase not explicitly represented.

The sensitivity analysis demonstrates that parameters related to egg compartment (such as o(T), $v_{t}$, $s_{a}\left(T\right)$ and $\mu_{e}$) did not play a strong role in model uncertainty. When tracking the egg population, it is clear that an abrupt growth occurs, forcing the adult population quickly towards its carrying capacity, even when its parameters are underestimated (Figure S1 in Supplementary Materials File S1). It is noteworthy, however, that this growth behavior demonstrates the relevance of an explicit egg compartment in the model.

Rainfall influence on epidemic peaks is related to the timing of egg hatching. Low rainfall rates can bring the mosquito adult population size to its carrying capacity, but cannot alter the general dengue impact. Other aspects of mosquito biology such as eggs, larvae and pupae mortality and oviposition rates, are also influenced by rainfall rates [61]. Although the presented model did not assess this impact, this should be a concern in future studies if the model should be improved.

The last investigated parameter was vertical transmission. Most dengue transmission models do not include vertical transmission [11,34], as it complicates the model too much and it is assumed it would not bring significant changes in the model output. However, in this study, vertical transmission played an important role. When removed, it altered the transmission dynamics, modifying the timing of the dengue epidemic peaks and the overall burden. The effect of different transmission values was assessed as indicated in Figure 6, and the higher the vertical transmission, the higher the overall burden calculated by the attack rate in the simulations. This finding suggests the need to further investigate the contribution of vertical transmission to dengue transmission dynamics, and more studies are required concerning the biology of the vector and its interaction with the virus to benefit future models.

The introduced framework highlighted the importance of tracking the egg compartment of the mosquito population, including vertical transmission and rainfall dependence. Overall, the simulations indicate similar patterns to observed data. Future assessments should include further improvements, such as the inclusion of the four dengue serotypes, simplifying the model by removing parameters that did not affect the model results and curves shape, adapting variables for each municipality environment, adding compartments for aquatic mosquito stages, and improving rainfall dependency and vertical transmission.

## Figures and Tables

**Table 1:** Constant model parameters. ${}^{†}$ these values were obtained from the literature as daily rates and were converted to 8 day${}^{-1}$. ${}^{‡}$ converted from annual rates.

Parameter	Definition	Value	Source
$f_{v}$	Mosquito sex ratio	0.5	[26]
$\mu_{e}$	Egg mortality rate (8 day)${}^{-1}$	0.077255 ${}^{†}$	[3]
$v_{t}$	Vertical transmission rate	0.1	[21]
γ	Dengue recovery rate (8 day)${}^{-1}$	1	[33]
$i_{m}$	Infected immigrants	0.001	
$\mu_{h}$	Human birth and mortality rate (8 day)${}^{-1}$	0.0003656 ${}^{‡}$	[27]
*K*	Carrying Capacity	0.7	[31]

**Table 2:** Temperature- and rainfall-dependent responses for *Aedes aegypti* biological traits. Traits were noted as Brière $\left[aT\left(T-b\right){\left(c-T\right)}^{\frac{1}{2}}\right]$ or quadratic [a(T−b)(T−c)], $\left[a\left(R^{2}+bR\right)\right]$ functions, where *T* represents temperature and R represents rainfall.

Variable	Definition	Function	*a*	*b*	*c*	Source
a(T)	Biting rate (8 day)${}^{-1}$	Brière	0.00161	13.35	40.08	[34]
o(T)	Oviposition rate per 8 days	Brière	0.06848	14.58	34.61	[34]
$s_{a}\left(T\right)$	Aquatic survival rate	Quadratic	−0.00599	13.56	38.29	[34]
$p_{hm}\left(T\right)$	human to mosquito infection prob. per bite	Brière	0.000491	12.22	37.46	[34]
$p_{mh}\left(T\right)$	mosquito to human infection prob. per bite	Brière	0.000849	17.05	35.83	[34]
$\mu_{v}\left(T\right)$	Adult mosquito mortality rate (8 day)${}^{-1}$	Quadratic	−0.0185	9.16	37.73	[34]
d(R)	Development rate	Quadratic	−2.29574	−1.18161		[36]

**Table 3:** Initial municipality-specific conditions for simulations.

Municipality	$N_{H}$	$S_{H}\left(0\right)$	$I_{H}\left(0\right)$	Tourists (8 day)${}^{-1}$
Rio de Janeiro	6320.446	327,259	40	21,535
Fortaleza	2452.185	87.734	70	2099
Porto Alegre	1409.351	171,628	2	14,325
Foz do Iguaçu	256,088	37,332	35	15,892

**Table 4:** Adapted parameter values resulting from the exploratory analysis in four different geographical contexts. Concerning the temperature and rainfall dependent functions, the new value substitutes the *a* variable within their function.

Municipality	$p_{\mathrm{hm}}\left(T\right)$	$p_{\mathrm{mh}}\left(T\right)$	a(T)	*K*	d(R)
Rio de Janeiro	0.000491	0.0003396	0.00161	0.6	−0.1607018
Fortaleza	0.0001473	0.0003396	0.0012075	3	−0.229574
Porto Alegre	0.0002946	0.0002547	0.000966	0.6	−0.688722
Foz do Iguaçu	0.000491	0.0001698	0.00161	3	−0.0114787

**Table 5:** Observed and simulated attack rates (%) by municipality.

Municipality	Observed Attack Rate (%)	Simulated Attack Rate (%)
Rio de Janeiro	6.52	14.57
Fortaleza	10.34	16.36
Porto Alegre	0.37	0.46
Foz do Iguaçu	35.96	26.32

## Data Availability

Dengue cases data are available at https://info.dengue.mat.br/, last accessed in 15 May 2021. Temperature data are available at https://developers.google.com/earth-engine/datasets/catalog/MODIS_006_MOD11A1, last accessed in 15 May 2021. Rainfall data are available at https://developers.google.com/earth-engine/datasets/catalog/UCSB-CHG_CHIRPS_DAILY, last accessed in 15 May 2021.

## Supplementary Materials

The following are available online at https://www.mdpi.com/article/10.3390/ijerph18189493/s1, Figure S1: Simulations of adult *Aedes aegypti* populations from 2010 to 2019 for Fortaleza, Foz de Iguaçu, Porto Alegre and Rio de Janeiro municipalities, Figure S2: Brieré equation curve: $2.59R\left(R-0\right)({\left(1-R\right)}^{\frac{1}{2}})$, Figure S3: Association between egg eclosion rate (day${}^{-8}$) and rainfall represented by the quadratic function: $-2.29574834*R^{2}+b*2.71268315$, Figure S4: Average temperature per 8 days from 2010 to 2019 in Fortaleza (blue), Foz de Iguaçu (orange), Porto Alegre (green) and Rio de Janeiro (red) municipalities, Brazil, Figure S5: Mean rainfall per 8 days from 2010 to 2019 in Fortaleza (blue), Foz de Iguaçu (orange), Porto Alegre (green) and Rio de Janeiro (red), Brazil, Figures S6–S9: first, second and total order sensitivity analyses: interactions between $O_{t}\left(T\right)$ (0.5–2), $v_{t}$ (0–0.3), *K* (0.5–3), a(T) (0.5–2), $i_{m}$ (0.00001–0.01), $p_{mh}$ (0.5–2), $p_{hm}$ (0.5–2), $f_{v}$ (0.3–0.7), $u_{e}$ (0.01–0.15), $u_{v}$ (0.3–0.7), γ (0.4–1.6), $u_{h}$ (0.00001–0.001), sa(T) (0.5–2) and model output sum of square errors (SSE), Figures S10–S13: Sensitivity Analysis residues regarding the sum of square errors (SSE) from model simulations and simulation parameters with their respective range of possibilities: $O_{t}\left(T\right)$ (0.5–2), $v_{t}$ (0–0.3), *K* (0.5, 3), a(T) (0.5–2), $i_{m}$ (0.00001–0.01), $p_{mh}$ (0.5–2), $p_{hm}$ (0.5–2), $f_{v}$ (0.3–0.7), $u_{e}$ (0.01–0.15), $u_{v}$ (0.3–0.7), γ (0.4–1.6), $u_{h}$ (0.00001–0,001), sa(T) (0.5–2), Figure S14: Chikungunya incidence in Fortaleza from 2010 to 2020 according to Infodengue data, Figure S15: Zika incidence in Rio de Janeiro from 2010 to 2020 according to Infodengue data.
