# Supplementary material for: A Framework for Weather-Driven Dengue Virus Transmission Dynamics in Different Brazilian Regions
Source: Int J Environ Res Public Health. 2021 Sep 9;18(18):9493. doi: 10.3390/ijerph18189493 (PMC8466780; doi:10.3390/ijerph18189493)
Supplement: Supplementary file 1 [file ijerph-18-09493-s001.zip › ijerph-1292835-supplementary.pdf]

Supplementary Materials: A Framework for Weather-Driven Dengue Virus Transmission Dynamics in Different Regions of Brazil

Leon D. Alves, Raquel M. Lana and Flávio C. Coelho

This supplementary materail complements the article "A framework for weather-driven DENV transmission dynamics in different regions of Brazil". All the Figures were created using the Matplotlib library of Python 3 programming language.

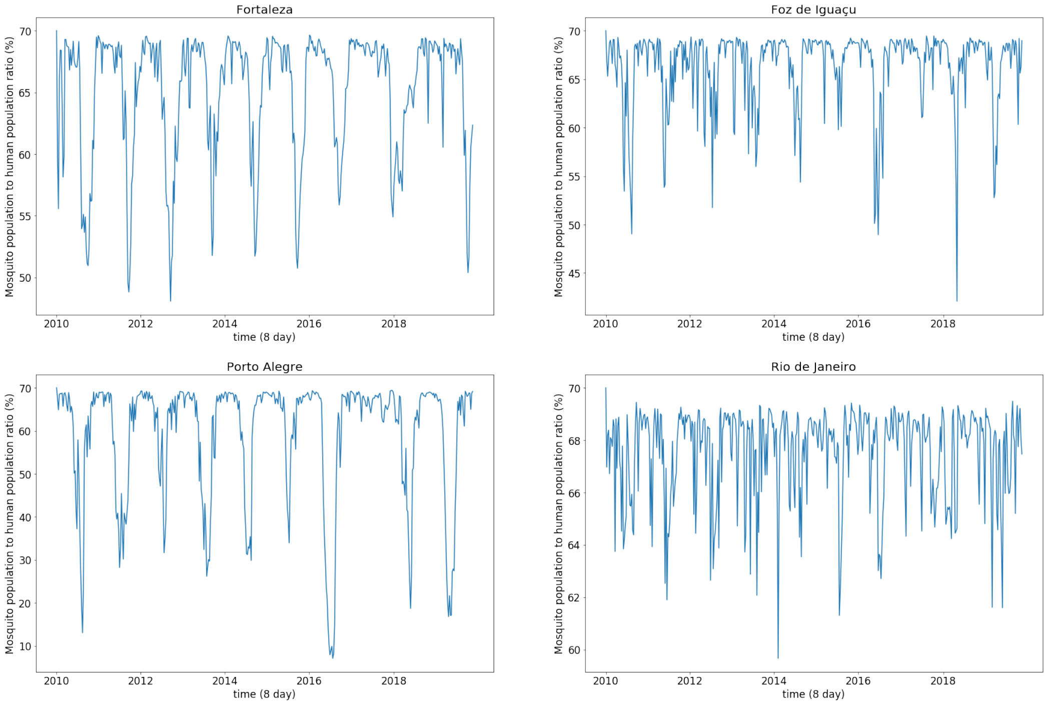

Figure S1. Simulations of adult populations of Aedes aegypti from 2010 to 2019 for Fortaleza, Foz de Iguaçu, Porto Alegre and Rio de Janeiro municipalities.

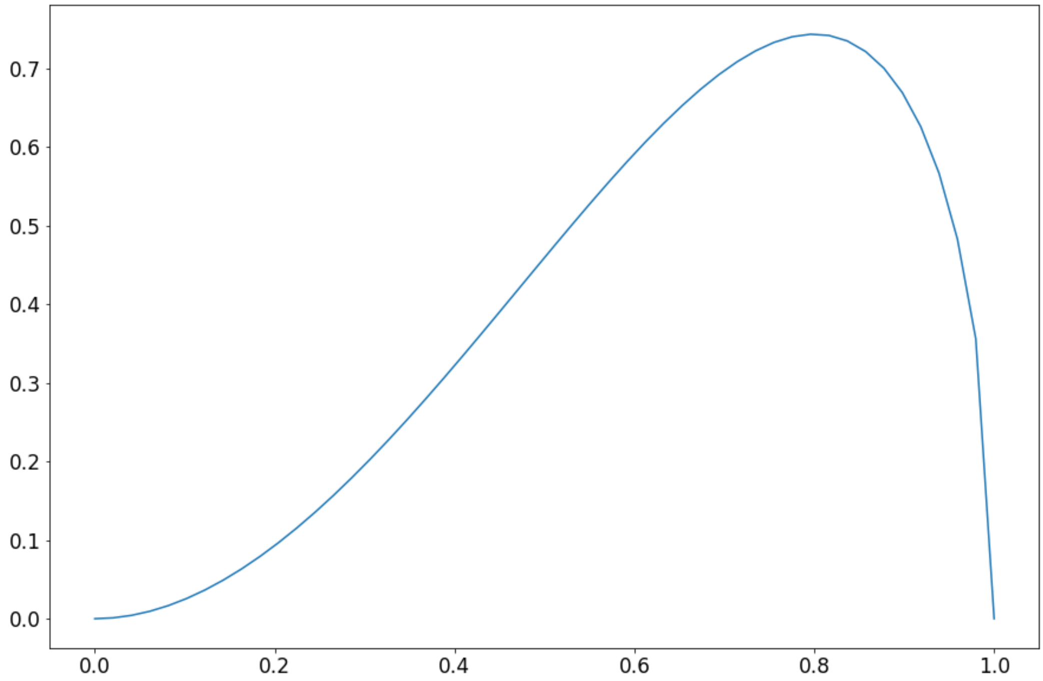

Figure S2. Brieré curve of the equation: $2.59R(R-0)((1-R)^{\frac{1}{2}})$

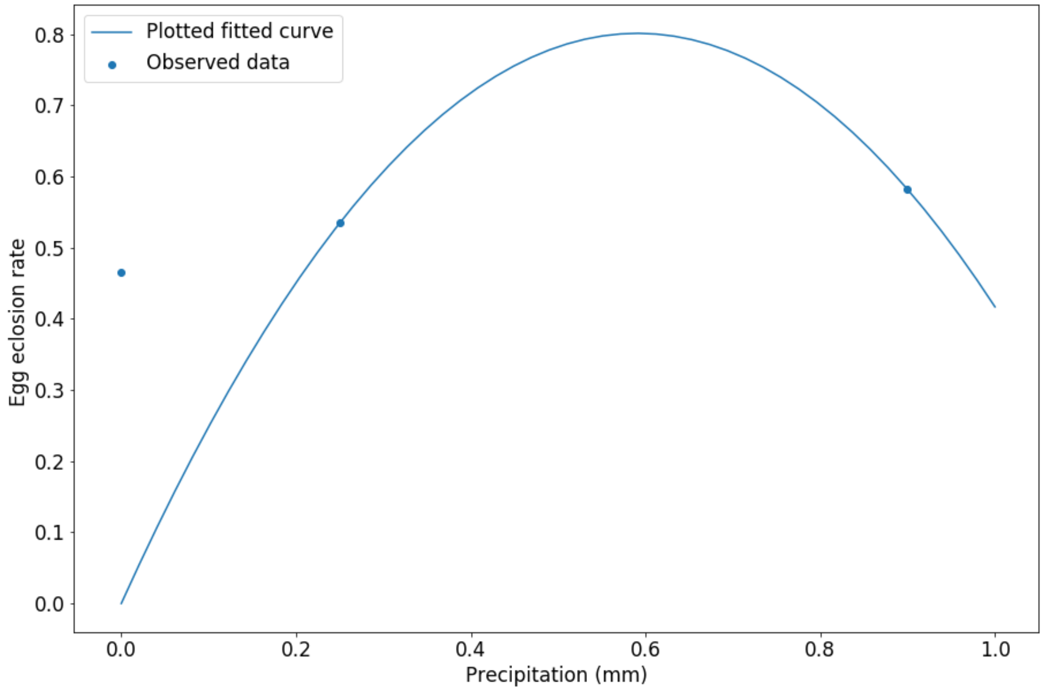

Figure S3. Relation between egg eclosion rate (day^{-8}) and rainfall represented by the quadratic function: $-2.29574834 * R^2 + b * 2.71268315$.

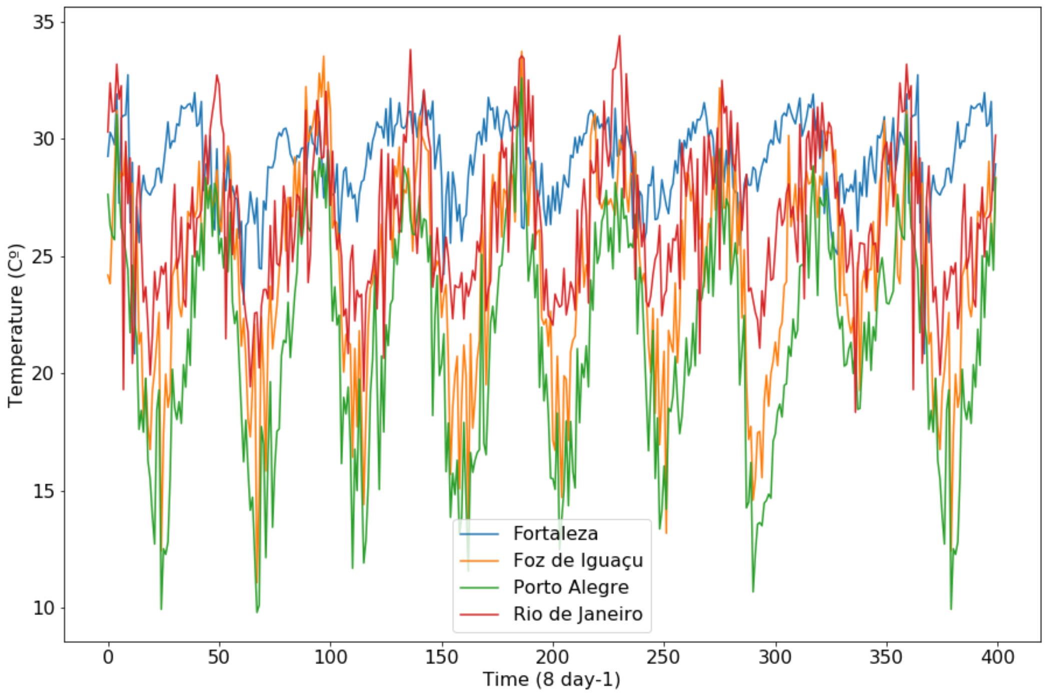

Figure S4. Average temperature per 8 days from 2010 to 2019 in Fortaleza (Blue), Foz de Iguaçu (Orange), Porto Alegre (Green) and Rio de Janeiro (Red) municipalities, Brazil.

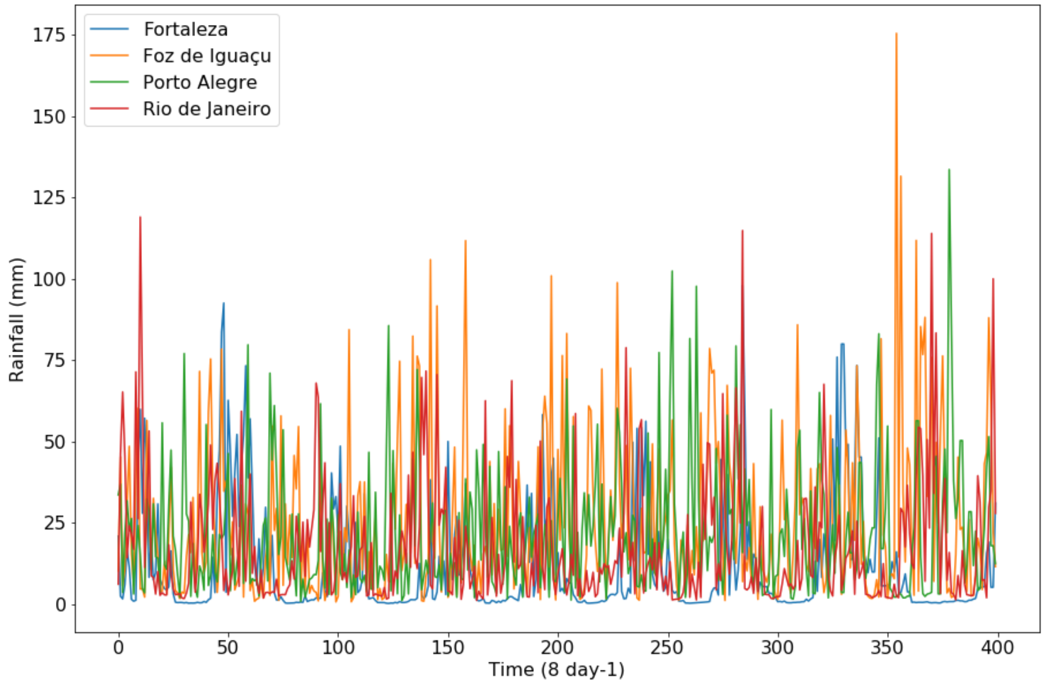

Figure S5. Mean rainfall per 8 days from 2010 to 2019 in Fortaleza (Blue), Foz de Iguaçu (Orange), Porto Alegre (Green) and Rio de Janeiro (Red) municipalities, Brazil.

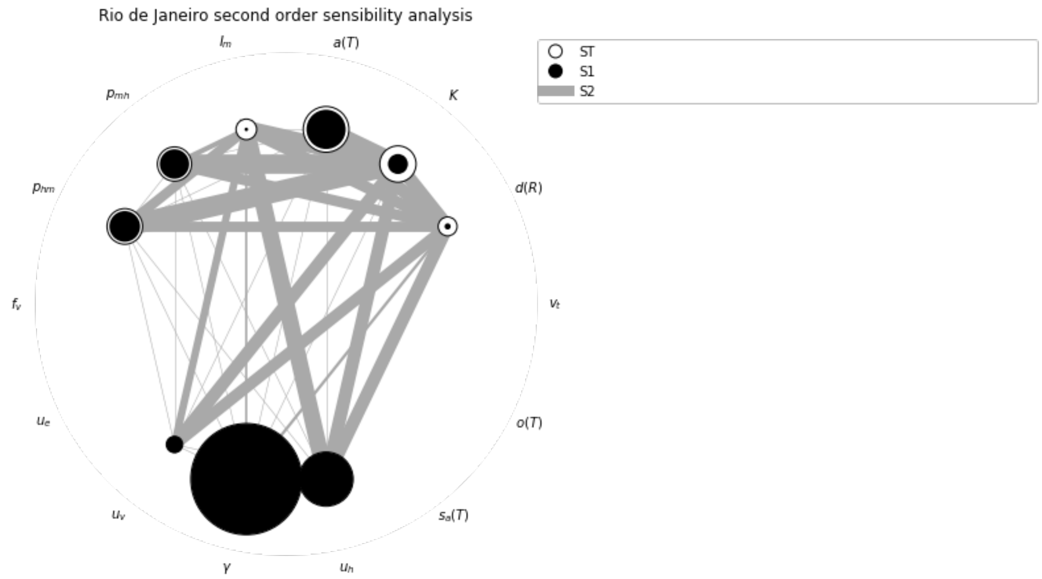

Figure S6. Porto Alegre first, second and total order sensitivity analysis: interaction between $O_t(T)$(0.5-2), v_t(0-0.3), K (0.5, 3), $a(T)$ (0.5-2), i_m (0.00001-0.01), p_{mh} (0.5-2), p_{hm} (0.5-2), f_v (0.3-0.7), u_e (0.01-0.15), u_v (0.3-0.7), γ (0.4-1.6), u_h (0.00001-0,001), $sa(T)$ (0.5-2) and model output sum of square errors (SSE).

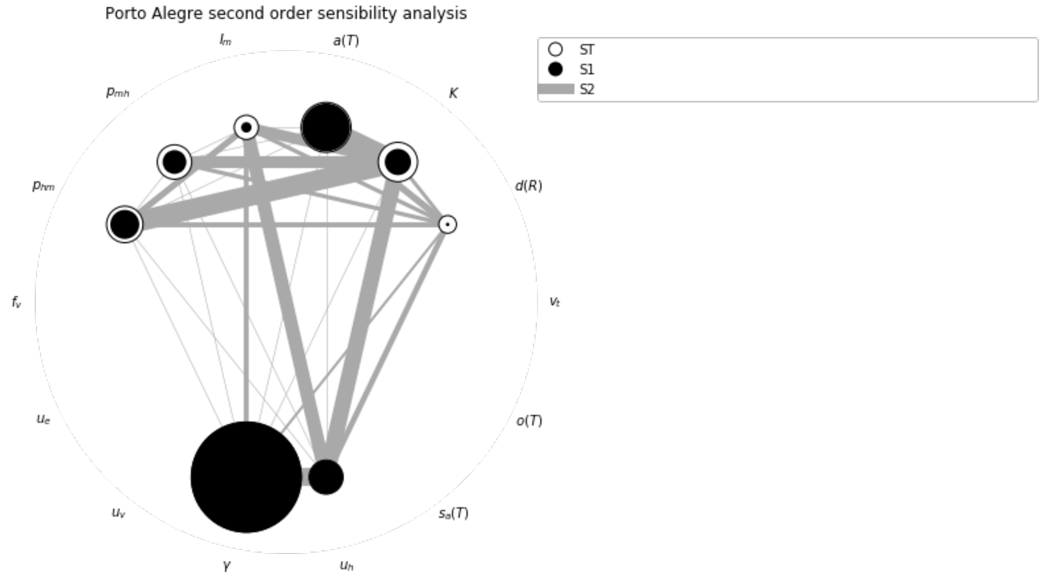

Figure S7. Rio de Janeiro first, second and total order sensitivity analysis: interaction between $O_t(T)$(0.5-2), v_t(0-0.3), K (0.5, 3), $a(T)$ (0.5-2), i_m (0.00001-0.01), p_{mh} (0.5-2), p_{hm} (0.5-2), f_v (0.3-0.7), u_e (0.01-0.15), u_v (0.3-0.7), γ (0.4-1.6), u_h (0.00001-0,001), $sa(T)$ (0.5-2) and model output sum of square errors (SSE).

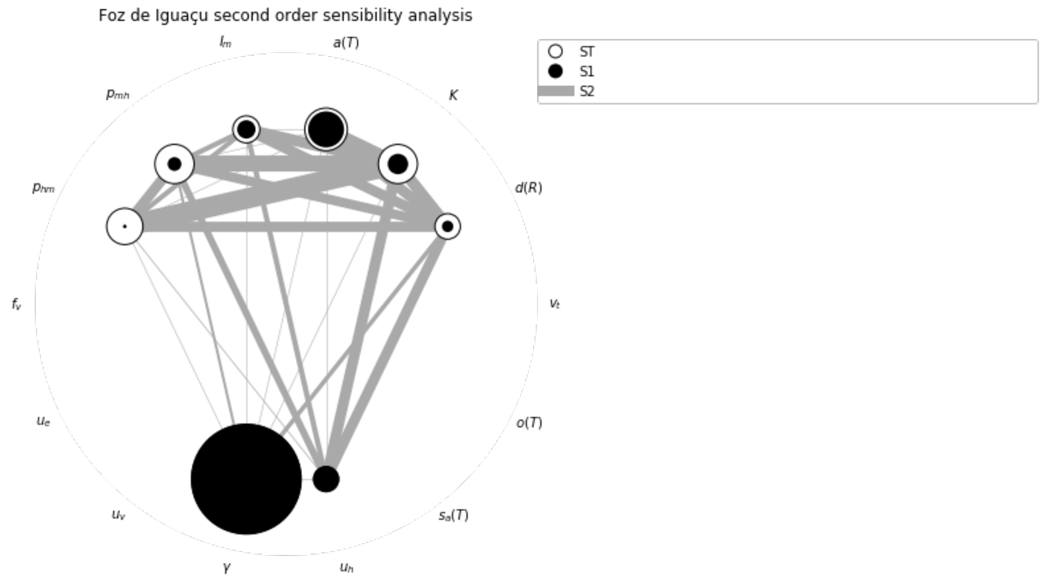

Figure S8. Foz de Iguaçu first, second and total order sensitivity analysis: interaction between $O_t(T)$(0.5-2), v_t(0-0.3), K (0.5, 3), $a(T)$ (0.5-2), i_m (0.00001-0.01), p_{mh} (0.5-2), p_{hm} (0.5-2), f_v (0.3-0.7), u_e (0.01-0.15), u_v (0.3-0.7), γ (0.4-1.6), u_h (0.00001-0,001), $sa(T)$ (0.5-2) and model output sum of square errors (SSE).

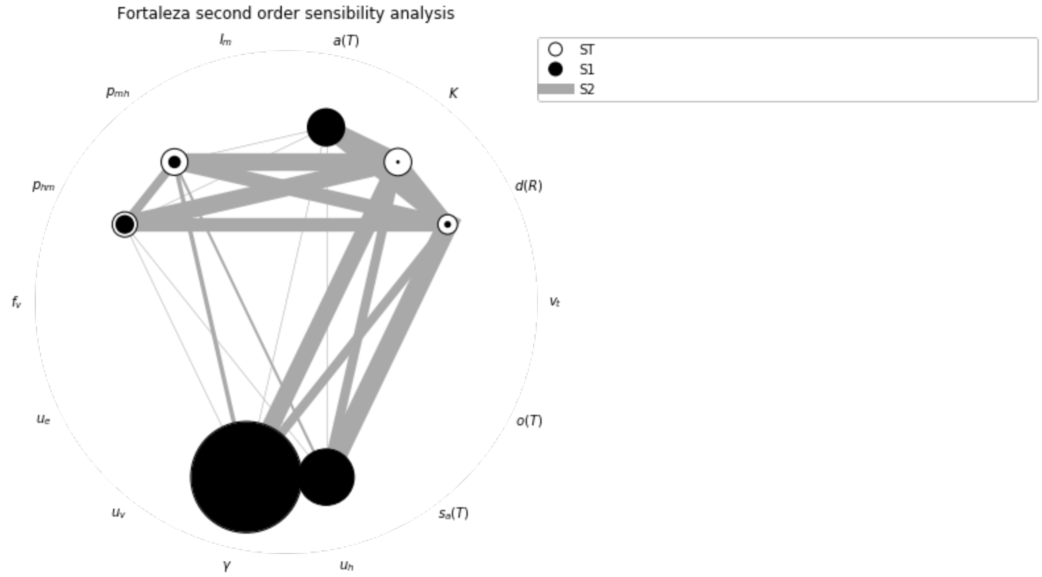

Figure S9. Fortaleza first, second and total order sensitivity analysis: interaction between $O_t(T)$(0.5-2), v_t(0-0.3), K (0.5, 3), $a(T)$ (0.5-2), i_m (0.00001-0.01), p_{mh} (0.5-2), p_{hm} (0.5-2), f_v (0.3-0.7), u_e (0.01-0.15), u_v (0.3-0.7), γ (0.4-1.6), u_h (0.00001-0,001), $sa(T)$ (0.5-2) and model output sum of square errors (SSE)

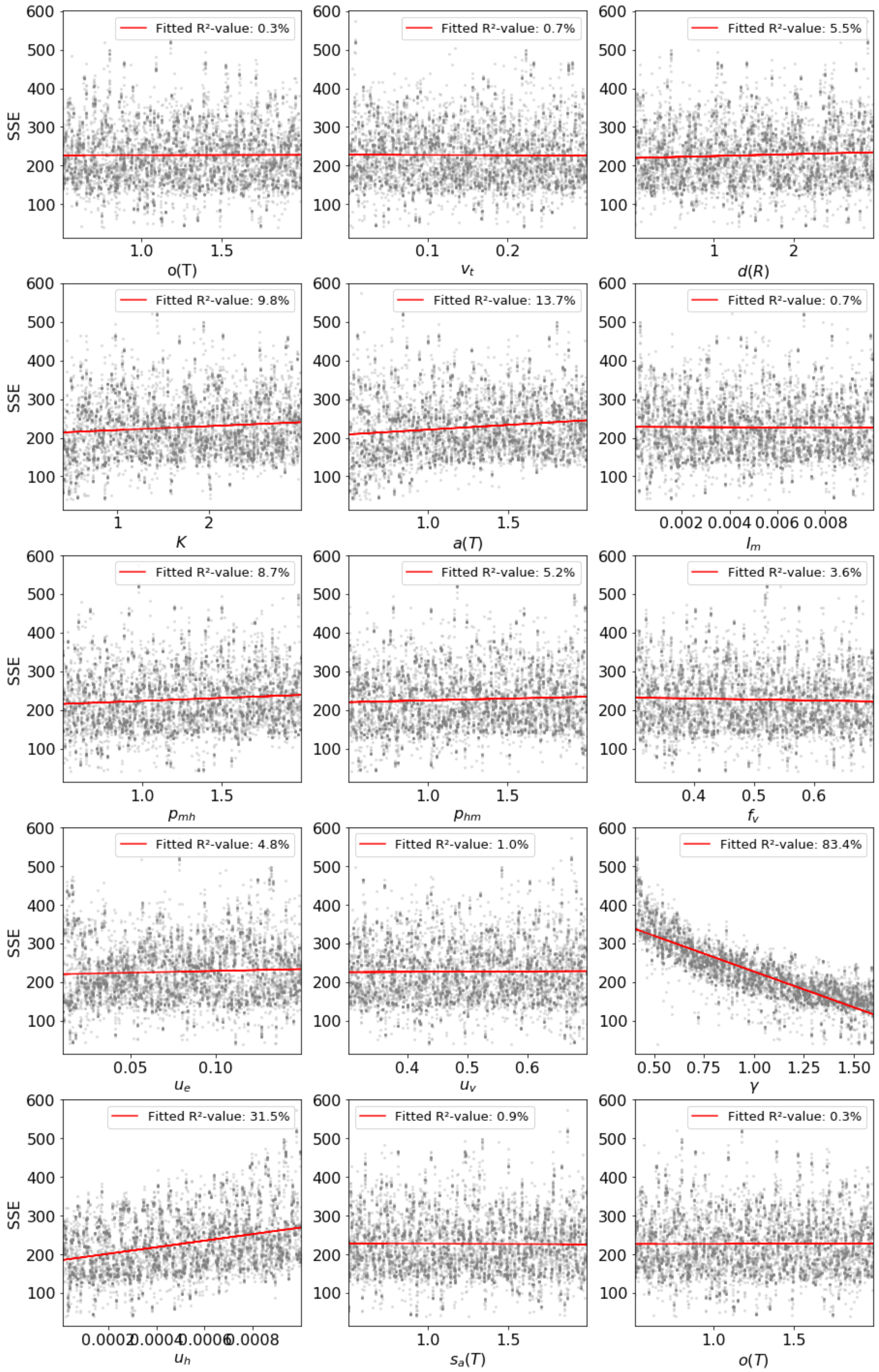

Figure S10. Sensitivity Analysis residues regarding sum of square errors (SSE) from model simulations and simulations parameters with the respecting range of possibilities: $O_t(T)$(0.5-2), v_t(0-0.3), K (0.5, 3), $a(T)$ (0.5-2), i_m (0.00001-0.01), p_{mh} (0.5-2), p_{hm} (0.5-2), f_v (0.3-0.7), u_e (0.01-0.15), u_v (0.3-0.7), γ (0.4-1.6), u_h (0.00001-0,001), $sa(T)$ (0.5-2) ; for Rio de Janeiro

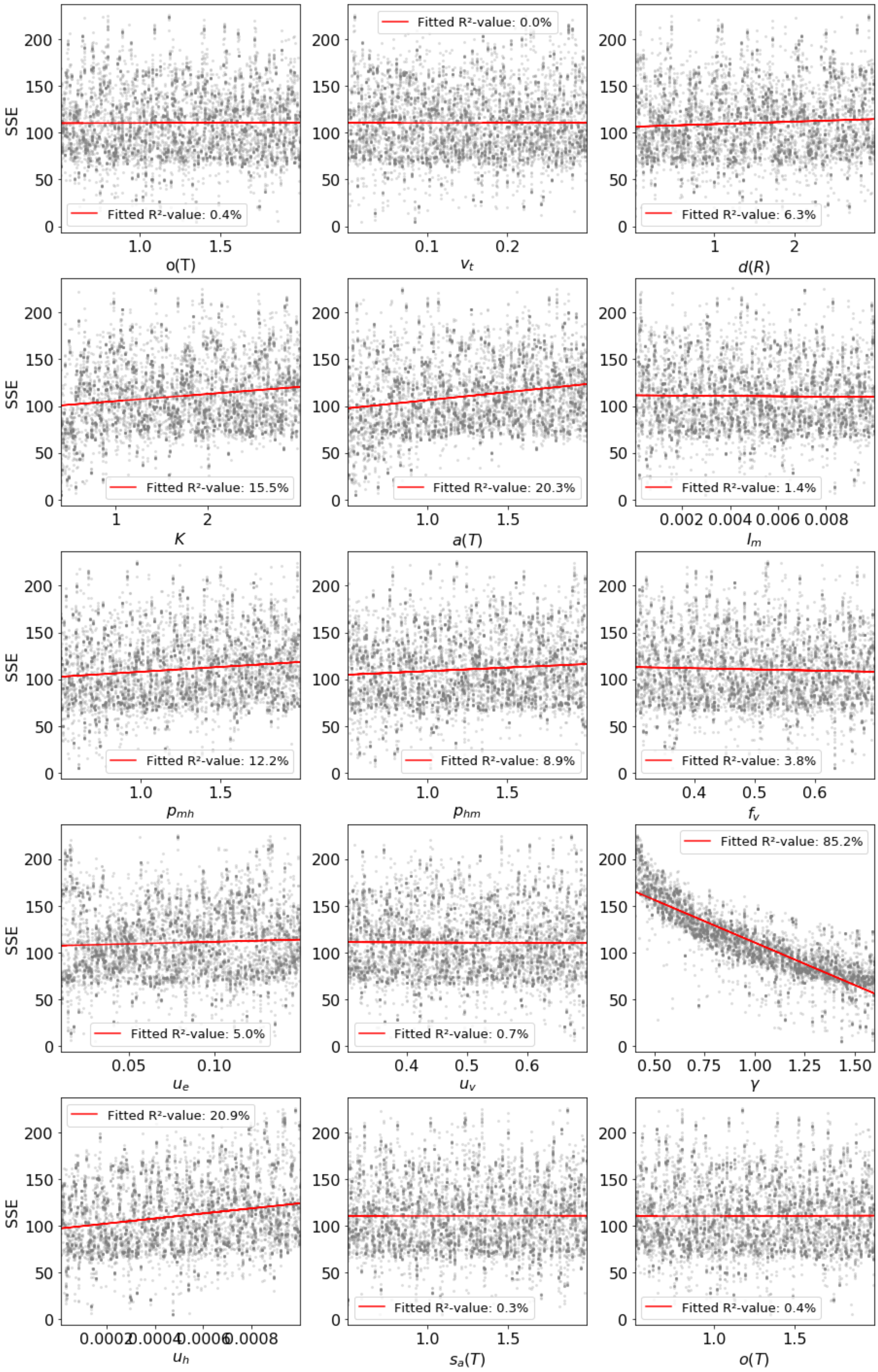

Figure S11. Sensitivity Analysis residues regarding sum of square errors (SSE) from model simulations and simulations parameters with the respecting range of possibilities: $O_t(T)$(0.5-2), v_t(0-0.3), K (0.5, 3), $a(T)$ (0.5-2), i_m (0.00001-0.01), p_{mh} (0.5-2), p_{hm} (0.5-2), f_v (0.3-0.7), u_e (0.01-0.15), u_v (0.3-0.7), γ (0.4-1.6), u_h (0.00001-0,001), $sa(T)$ (0.5-2) ; for Porto Alegre

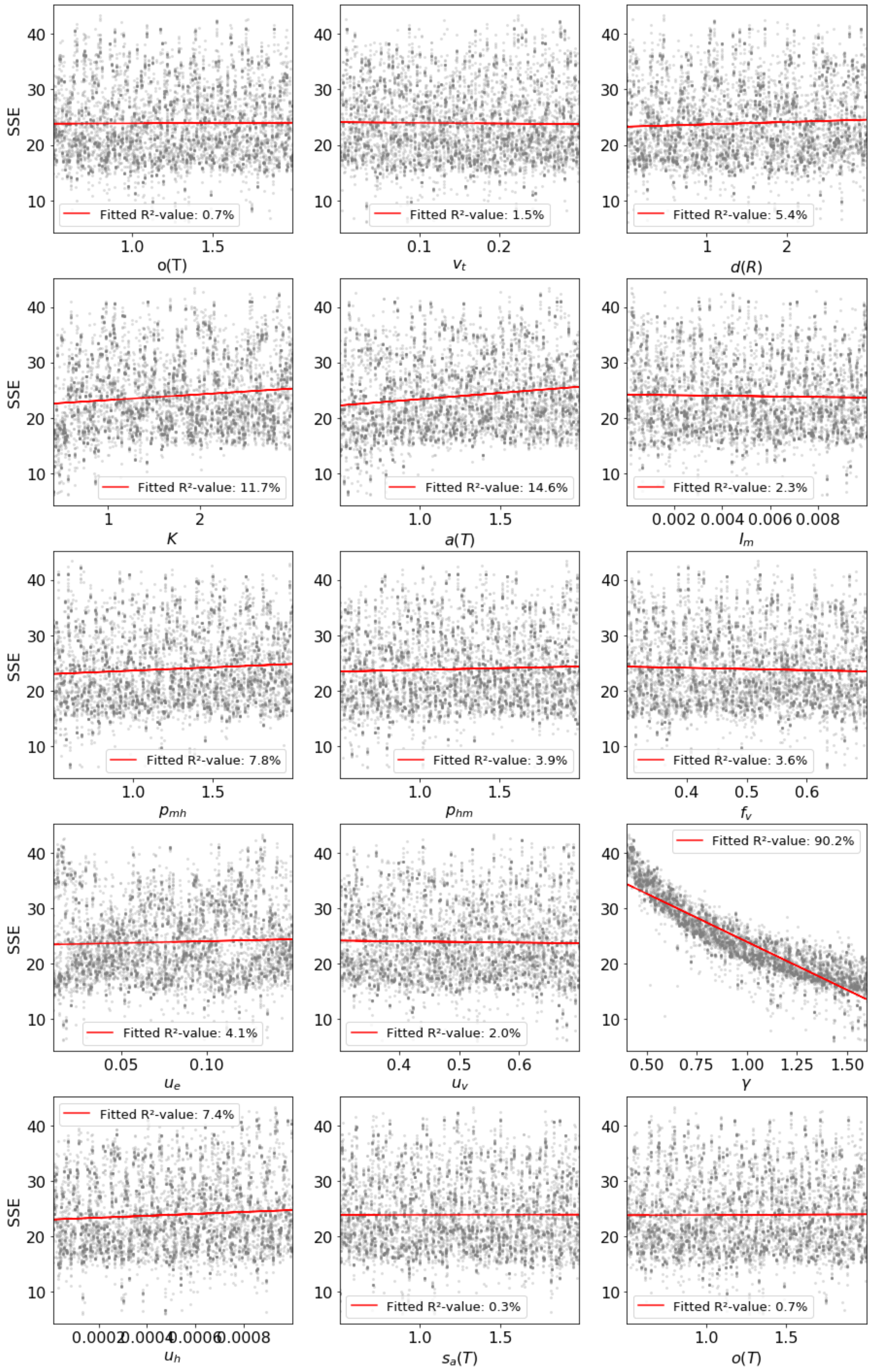

Figure S12. Sensitivity Analysis residues regarding sum of square errors (SSE) from model simulations and simulations parameters with the respecting range of possibilities: $O_t(T)$(0.5-2), v_t(0-0.3), K (0.5, 3), $a(T)$ (0.5-2), i_m (0.00001-0.01), p_{mh} (0.5-2), p_{hm} (0.5-2), f_v (0.3-0.7), u_e (0.01-0.15), u_v (0.3-0.7), γ (0.4-1.6), u_h (0.00001-0,001), $sa(T)$ (0.5-2) ; for Foz de Iguaçu

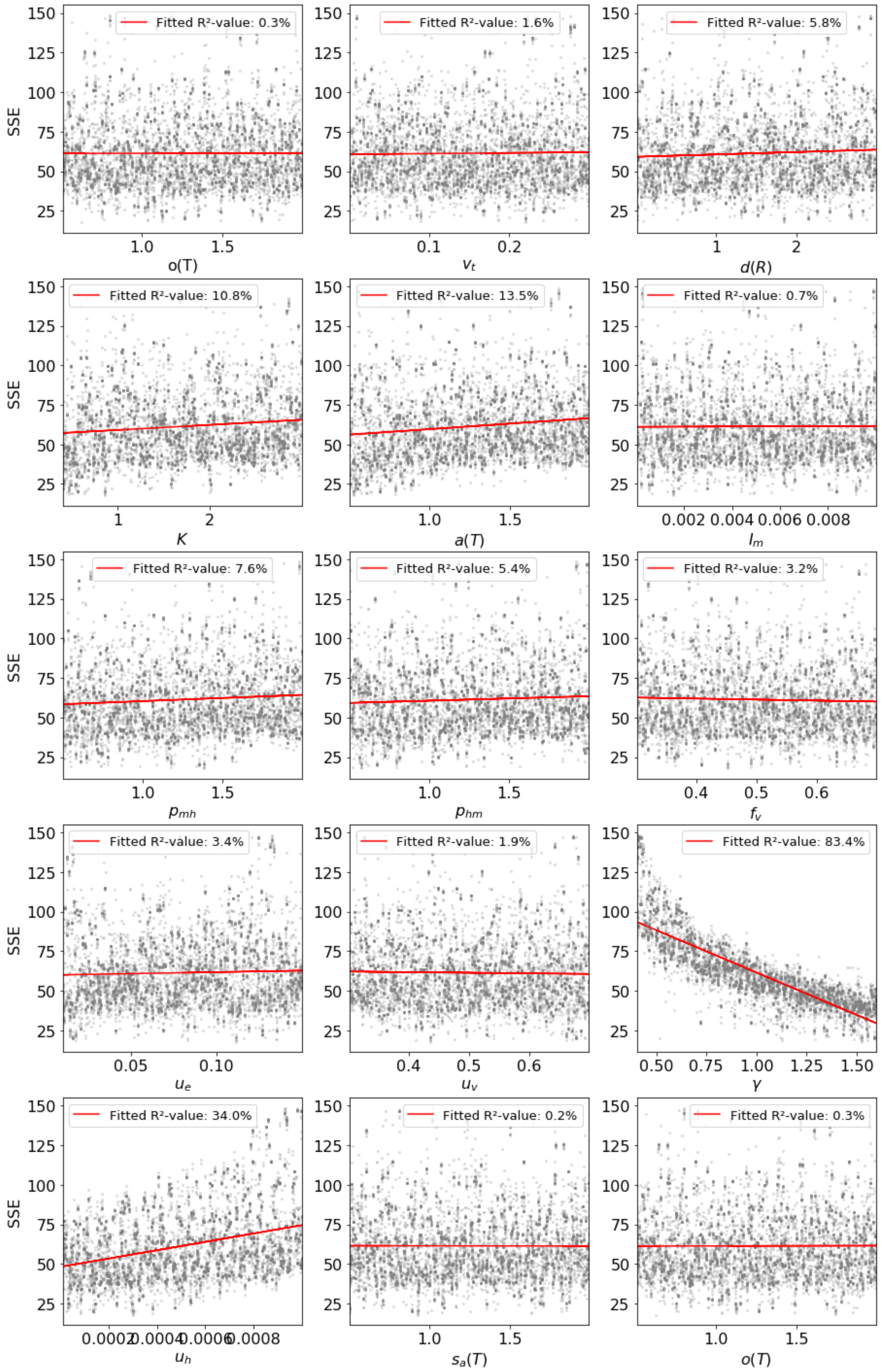

Figure S13. Sensitivity Analysis residues regarding sum of square errors (SSE) from model simulations and simulations parameters with the respecting range of possibilities: $O_t(T)$(0.5-2), v_t(0-0.3), K (0.5, 3), $a(T)$ (0.5-2), i_m (0.00001-0.01), p_{mh} (0.5-2), p_{hm} (0.5-2), f_v (0.3-0.7), u_e (0.01-0.15), u_v (0.3-0.7), γ (0.4-1.6), u_h (0.00001-0,001), $sa(T)$ (0.5-2) ; for Fortaleza

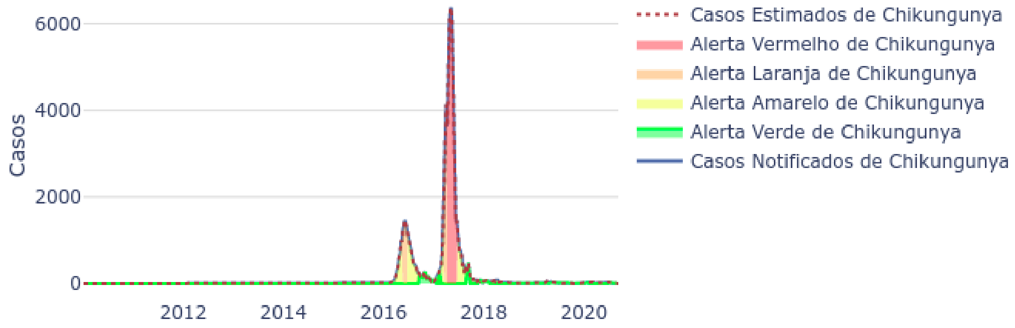

Figure S14. Chikungunya incidence in Fortaleza from 2010 to 2020 according to Infodengue data

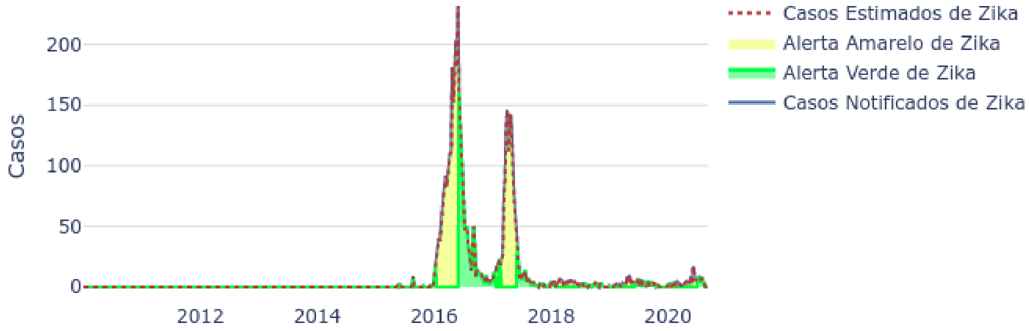

Figure S15. Zika incidence in Rio de Janeiro from 2010 to 2020 according to Infodengue data
